# Investigation of Zinc and Phosphorus Elements Incorporated into Micro-Arc Oxidation Coatings Developed on Ti-6Al-4V Alloys

**DOI:** 10.3390/ma11030344

**Published:** 2018-02-27

**Authors:** Yaping Wang, Lilan Zeng, Honghua Zhang, Junhuai Xiang, Shufang Zhang, Wenhui Chang, Rongfa Zhang, Qiao Wang, Yang Sheng, Ying Zhao

**Affiliations:** 1 School of Materials and Electromechanics, Jiangxi Science and Technology Normal University, Nanchang 330013, China; 18679934872@163.com (Y.W.); zhanghonghua@impcas.ac.cn (H.Z.); xiangjunhuai@163.com (J.X.); zhangshufanglaoshi@163.com (S.Z.); 13647089261@163.com (W.C.); qiao15879167182@163.com (Q.W.); 18379921096@163.com (Y.S.); 2 Shenzhen Institutes of Advanced Technology, Chinese Academy of Sciences, Shenzhen 518055, China; ll.Zeng@siat.ac.cn

**Keywords:** titanium alloys, micro-arc oxidation, zinc ions, mechanism

## Abstract

In order to clarify the mechanism that zinc and phosphorus elements entering the micro-arc oxidation (MAO) coatings developed on Ti-6Al-4V alloys, anodic coatings containing different zinc and phosphorus were fabricated using an orthogonal experiment of four factors with three levels in an electrolyte containing EDTA-ZnNa_2_, KOH, and phytic acid. Surface morphology, element composition, chemical state and phase structure of MAO coatings were characterized by scanning electron microscope (SEM), energy dispersive X-ray spectrometer (EDS), X-ray photoelectron spectroscopy (XPS) and X-ray diffraction (XRD). The concentrations of zinc and phosphorus in the electrolyte were analyzed by an inductively coupled plasma optical emission spectrometry (ICP-OES). The results show that zinc and phosphorus elements in MAO coatings exist in the form of Zn_3_(PO_4_)_2_. Phytic acid is the most important factor on both zinc and phosphorus contents of MAO coatings. With the increase of phytic acid concentration or the decrease of KOH concentration, the contents of zinc and phosphorus in MAO coatings present a similarly increasing tendency. Our results indicate that phosphorus takes part in coating formation mainly by diffusion, while zinc enters into MAO coatings with phosphorus from phytic acid.

## 1. Introduction

Titanium alloys are widely used as metallic implants due to the combination of their outstanding characteristics such as high strength, low density, high immunity to corrosion, and good biocompatibility [1,2,3]. However, titanium alloys do not form sufficient osseointegration with surrounding bones in vivo [1,2] and also do not possess antibacterial ability [4]. Therefore, it is necessary to fabricate coatings with increased osseointegration and appropriate antibacterial properties on titanium alloys for clinic application [3,4].

**Figure 1:**
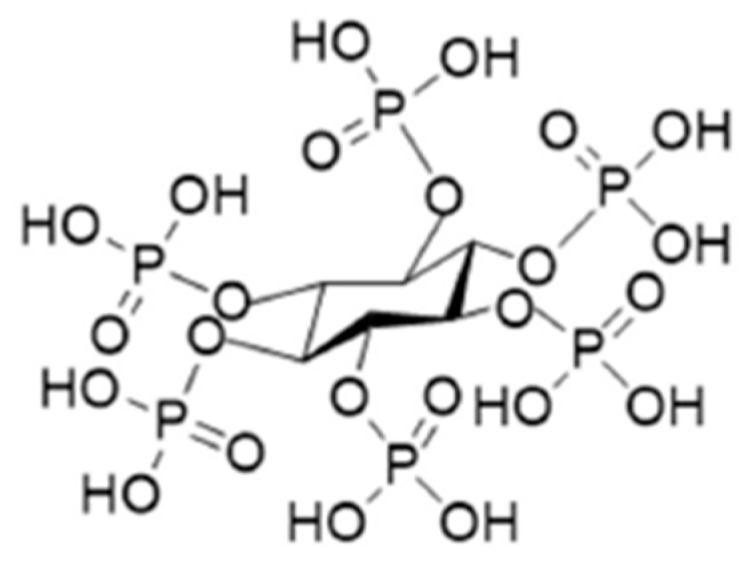
Chemical structure of phytic acid.

Among the various surface modification processes, micro-arc oxidation (MAO) has received considerable attention since the MAO coatings possess high hardness, good wear resistance, moderate corrosion resistance, and good thermal stability [3]. Especially, the functional coatings can be developed on titanium alloys by adjusting the electrolyte compositions, concentration and the applied electric parameters [3,5]. Phosphorus (P) is a main component of human bone, and phosphorus-containing anodic coatings on titanium implants can improve cell adhesion and proliferation [6]. Therefore, inorganic phosphorus-containing electrolytes, for example, H_3_PO_4_, glycerophosphate disodium salt pentahydrate, NaH_2_PO_4_, and (NaPO_3_)_6_ are widely used in MAO on titanium alloys [5,6,7,8,9]. As a natural and nontoxic organic macromolecule, phytic acid, also known as inositol hexakisphosphate (C_6_H_6_(PO_4_)_6_H_12_, as shown in Figure 1), is recently used as a novel phosphorus-containing electrolyte on titanium alloys and the fabricated MAO coatings show good biocompatibility [10].

Mn, Fe, Cu, Zn, and Se are considered as essential trace elements in humans [11]. For example, zinc (Zn) is involved in a great number of cellular processes, such as DNA synthesis, enzyme activity and cell division [7,11,12,13]. Moreover, Zn is one kind of inorganic antibacterial agent [7,8,11,12,13,14,15]. Therefore, it is important to fabricate MAO coatings with appropriate Zn content by using zinc-containing electrolytes like zinc acetate [7,14]. The results show that zinc-containing coatings are biocompatible for rat bone marrow stromal cells and present an increased proliferation and ALP expression [7]. However, the influencing regularity of processing factors on the Zn content in MAO coatings is rarely reported.

Cations and anions in water solution can enter into MAO coatings by diffusion, electric migration, and convection [16]. During MAO, an electric field developed between the anode and the cathode, becomes stronger with the increase of the applied current density or final voltage. Therefore, anions can arrive at the anode by both diffusion and electromigration, while cations move to the anode mainly by diffusion. In a solution containing chelating agents such as phytic acid, some cations, which have been combined with the chelating agents and become negatively charged, can enter MAO coatings by diffusion and electromigration [17]. At present, the effects of processing parameters on coating properties are widely investigated by the conventional method, namely, at a time, one factor is kept varying and all the other factors are kept constant. Compared with the conventional method, an orthogonal experiment can fast and accurately reveal the influences of processing factors on the objective parameters. Especially, the affecting sequence of processing factors, which is the most or the least important factor, can be achieved by the orthogonal experiment. Therefore, it is very meaningful to reveal the entrance mechanism of cations and anions into MAO coatings by using the orthogonal experiment.

In this work, ethylene diamine tetraacetic acid zinc disodium salt hydrate (C_10_H_12_N_2_O_8_Na_2_Zn, EDTA-ZnNa_2_), a zinc-containing soluble substance, was selected as the key composition of MAO electrolytes. The influences of EDTA-ZnNa_2_, KOH, phytic acid concentrations and treating time on the contents of Zn and P elements in MAO coatings were systematically investigated. The mechanism of Zn and P ions incorporated into MAO coatings was discussed as well.

## 2. Experimental

### 2.1. Materials and MAO Treatment

Ti-6Al-4V plates with the dimensions of 50 × 50 × 3 mm^3^ were used as the samples for MAO treatment. Samples were successively ground with SiC paper from 80 to 1000 grit, washed with distilled water and dried in a hot wind stream. The orthogonal experiment of four factors with three levels including EDTA-ZnNa_2_, KOH and phytic acid concentration as well as treating time was used to comprehensively investigate the effects of processing factors on the contents of Zn and P elements in MAO coatings.

The levels of each factor were selected according to the reported results and our previous experiments. The factors and levels of the orthogonal experiment are listed in Table 1.

**Figure 2:**
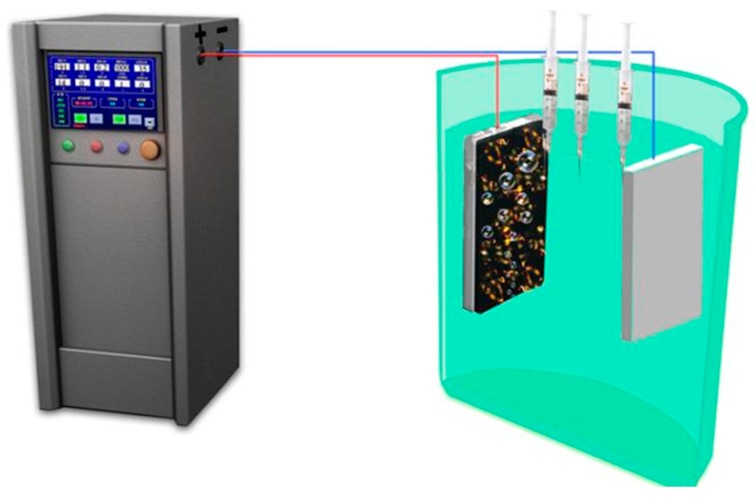
The schematic diagram of micro arc oxidation experiment.

EDTA-ZnNa_2_ and phytic acid were separately purchased from Shandong Xiya Reagent Chemistry Co. Ltd. (Linshu, China) and Sinopharm Chemical Reagent Co., Ltd. (Shanghai, China). A homemade MAO5D power supply (Chengdu Tongchuang New Material Surface Engineering and Technology Center, Chengdu, China) was used under a constant current control mode. For the power supply used, pulse frequency and duty cycle could be regulated in the range of 100–2000 Hz and 5–40%, respectively. A Ti-6Al-4V sample was placed into a plastic barrel and used as the anode, while another Ti-6Al-4V sample of 50 × 50 × 3 mm^3^ was used as the cathode, as shown in Figure 2. During MAO treatment, current density, pulse frequency, and duty cycle were kept constant. These were 110 mA/cm^2^, 2000 Hz, and 35%, respectively. The fabricated MAO samples containing 2.86 wt %, 5.83 wt %, and 8.81 wt % Zn were abbreviated as Zn-2.86 wt %, Zn-5.83 wt %, and Zn-8.81 wt %, respectively.

### 2.2. Measurement

#### 2.2.1. Surface Characterization

Surface morphology and chemical composition of the MAO-treated titanium alloys were characterized by a field emission scanning electron microscope (SEM, Zeiss, Oberkochen, Germany) and energy dispersive X-ray spectrometer (EDS) attached to SEM. The phase structure of the samples was analyzed by X-ray diffraction (XRD-6100, Shimadzu, Kyoto, Japan). An X-ray photoelectron spectroscopy (XPS, ESCALAB250, Thermo VG, Waltham, MA, USA) with an Al Kα anode (λ = 1486.6 eV) was used to detect the chemical states on the surface of MAO coatings.

#### 2.2.2. The Measurement of Zn and P concentration

In order to further clarify the underlying mechanism of Zn and P ions into MAO coatings, one MAO solution was simultaneously fetched by three syringes in anode area, the middle area and cathode area during treatment for 40 s and 120 s (Figure 2). The concentrations of Zn and P ions were analyzed by ICP-OES (PE Optima8300, Perkin-Elmer Corporation, Waltham, MA, USA).

## 3. Results

### 3.1. Results of the Orthogonal Experiment

The orthogonal experimental array and the contents of Zn and P elements analyzed by EDS were listed in Table 2.

The experimental data were treated by the method of the intuitionistic analysis. K_1_, K_2_ and K_3_ listed in Table 2 separately stand for the sum of Zn content in MAO coatings fabricated at Level 1, Level 2, and Level 3 of four factors. The maximum difference between the total Zn content at two levels of each factor shows the general influence of that factor. The greater the difference, the more important the factor. Based on this, the influencing sequence of factors on Zn content of MAO coating was ranked as phytic acid concentration > KOH concentration > EDTA-ZnNa_2_ concentration > treating time. With the increase of phytic acid concentration or the decrease of KOH concentration, the Zn content in MAO coatings increased. EDTA-ZnNa_2_ concentration was the third important affecting factor on the Zn content in MAO coatings. With the increasing EDTA-ZnNa_2_ concentration, the Zn content in MAO coatings increased from Level 1 to Level 2 but decreased from Level 2 to Level 3, indicating that the increased EDTA-ZnNa_2_ concentration could not continually increase the Zn content.

Table 2 also shows that among nine samples, sample No.7, namely fabricated in the solution of 10 g/L EDTA-ZnNa_2_, 2 g/L KOH and 8 g/L phytic acid, achieved the highest P content of 14.1 wt % in MAO coatings. The data of P content (listed in brackets) were dealt with the same method as the data of Zn content. The ranking of influencing factors on P content of anodic coatings was as follows: phytic acid concentration > KOH concentration > treating time> EDTA-ZnNa_2_ concentration.

**Figure 3:**
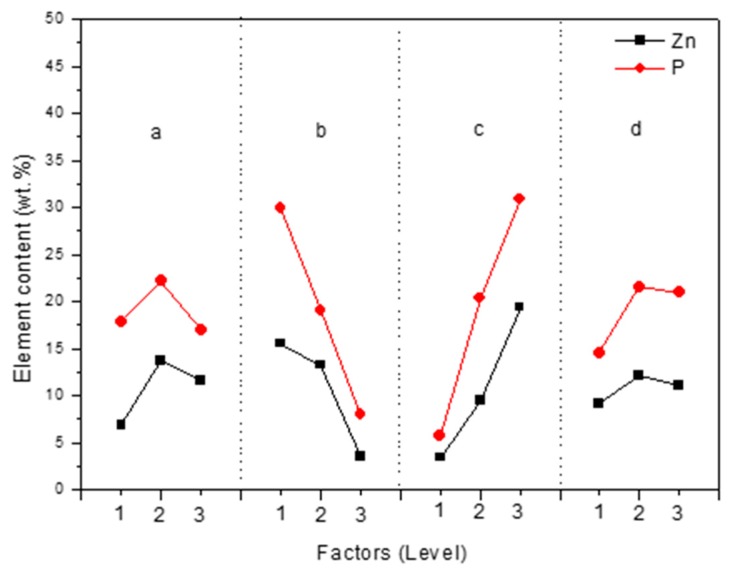
Effects of factors on the content of Zn and P elements in anodic coatings: (**a**) EDTA-ZnNa_2_ concentration; (**b**) KOH concentration; (**c**) phytic acid concentration; (**d**) treating time.

Based on the results listed in Table 2, the influences of the four factors on the Zn and P contents of MAO coatings were shown in Figure 3.

In the experiment, phytic acid concentration and KOH concentration were the main influencing factors on both Zn and P contents of anodic coatings (Table 2). In addition, their influencing regularity on Zn and P contents were similar. With the increasing phytic acid concentration or the decreasing KOH concentration, the contents of Zn and P in MAO coatings increased (Figure 3). With the increase of EDTA-ZnNa_2_ concentration or the prolonging of treating time, both Zn and P amounts firstly rapidly increased from Level 1 to Level 2, but then slightly decreased from Level 2 to Level 3. The results shown in Figure 3 indicated that the changing regularity of P amount in MAO coatings was similar to that of Zn amount.

### 3.2. Effects of Processing Factors on Coating Property

In order to reveal the influencing regularity of four factors on Zn and P contents, it is necessary to examine surface morphology, chemical composition, phase structure, and the element state of the coatings.

#### 3.2.1. Surface Morphology and Chemical Composition of the MAO Film

**Figure 4:**
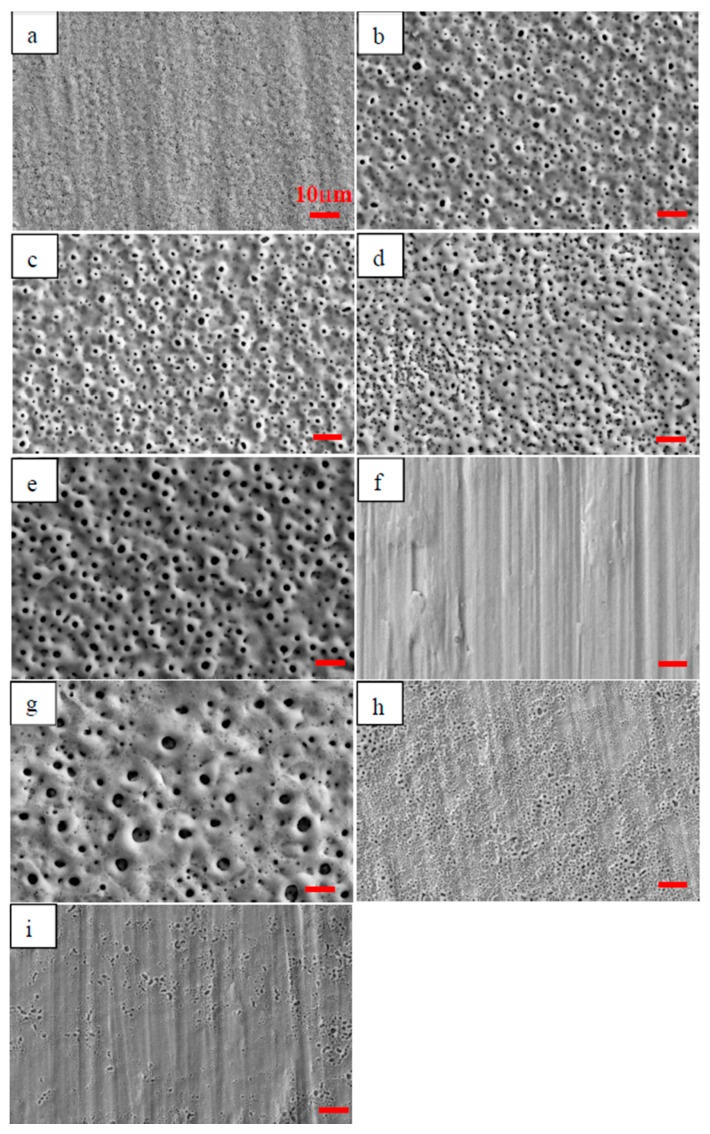
Surface morphologies of micro-arc oxidation (MAO) coatings developed under different processing parameters: (**a**) No. 1, (**b**) No. 2, (**c**) No. 3, (**d**) No. 4, (**e**) No. 5, (**f**) No. 6, (**g**) No. 7, (**h**) No. 8 and (**i**) No. 9.

As listed in Table 2, sample NO. 6 presented the lowest both Zn and P amount, while sample No. 7 achieved the highest both Zn and P amount. The surface morphologies of nine MAO coatings are shown in Figure 4.

As shown in Figure 4a, dense anodic coatings were formed on titanium alloys. In comparison, the coatings shown in Figure 4b–e and g exhibited a typically porous structure, suggesting that the MAO coatings were successfully developed. It was clearly that anodic coatings were not developed on sample No. 6 (Figure 4f). As shown in Figure 4h,i, porous anodic coatings were partially developed on samples No. 8 and No. 9. According to Figure 4 and the corresponding electrolyte composition, the MAO coatings were difficult to develop in solutions composed of low phytic acid concentration (Figure 4a,f,h) or high KOH concentration (Figure 4f,i).

#### 3.2.2. XRD Analysis

**Figure 5:**
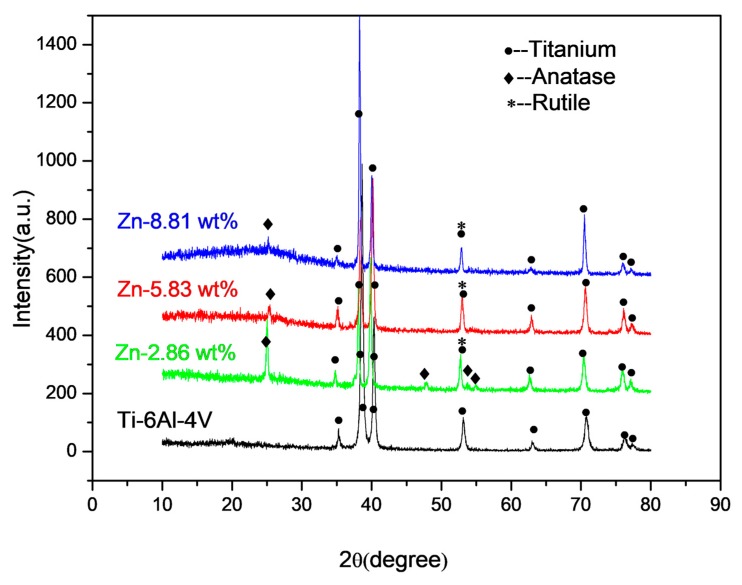
The X-ray diffraction patterns of the untreated Ti-6Al-4V, the MAO coatings containing 2.86 wt %, 5.83 wt % and 8.81 wt % zinc.

The XRD spectra of the untreated Ti6Al4V, Zn-2.86 wt %, Zn-5.83 wt % and Zn-8.81 wt % were shown in Figure 5.

As shown in Figure 5, the MAO coatings were mainly composed of anatase and rutile. No zinc-containing or phosphorus-containing substances were identified by XRD. Therefore, XPS was utilized to further characterize the chemical state of the elements in MAO coatings.

#### 3.2.3. XPS Analysis

**Figure 6:**
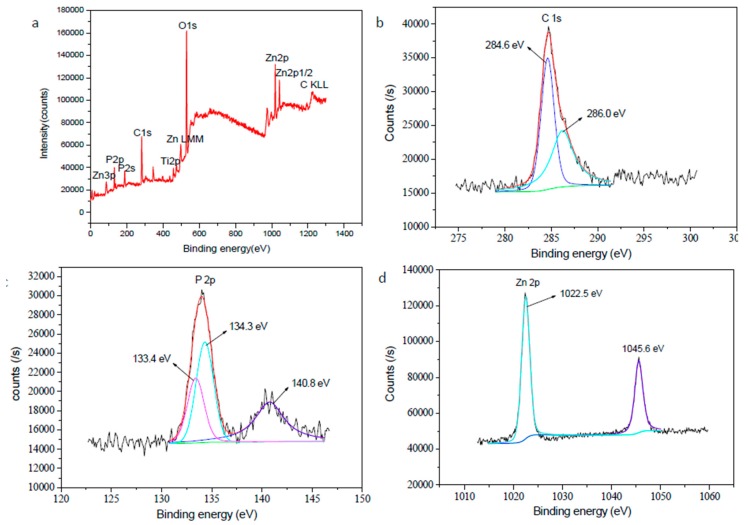
X-ray photoelectron spectroscopy (XPS) spectra of MAO coatings obtained under current density 50 mA/cm^2^, duty cycle 35%, pulse frequency 2000 Hz and treating time 3.0 min in the solution containing 10 g/L EDTA-ZnNa_2_, 2 g/L KOH and 8 g/L phytic acid: (**a**) survey; (**b**) C1s, (**c**) P 2p, (**d**) Zn 2p.

The survey spectrum of anodic coatings with Zn-8.81 wt % and the high-resolution XPS spectra of C, P and Zn elements are shown in Figure 6.

According to Figure 6a, C, O, Ti, P and Zn elements were present in MAO coatings. C 1s spectrum was divided into two peaks at binding energy of 284.6 and 286.0 eV (Figure 6b), separately corresponding to C–C or C–H and C–O [6]. The P 2p spectrum exhibited three peaks at 133.4, 134.3 and 140.8 eV, which were the phosphorus peaks of Zn_3_(PO_4_)_2_ [18]. The Zn 2p peaks at 1022.5 and 1045.6 eV were well assigned to Zn_3_(PO_4_)_2_ [18].

### 3.3. Zn and P Concentrations

**Figure 7:**
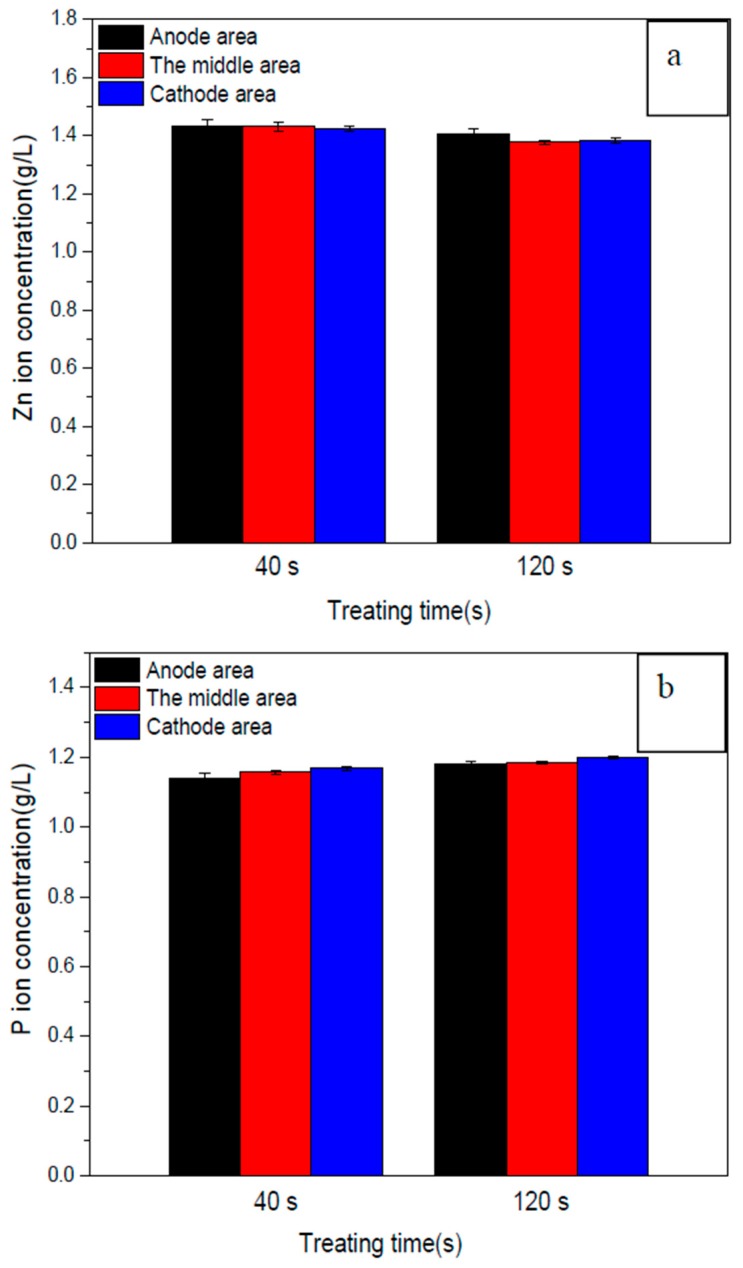
The concentrations of Zn and P in the solution containing 10 g/L EDTA-ZnNa_2_, 2 g/L KOH and 8 g/L phytic acid in anode area, the middle area and cathode area during MAO treatment for 40 s and 120 s.

For MAO times of 40 s and 120 s, the concentrations of Zn and P in the solution of 10 g/L EDTA-ZnNa_2_, 2 g/L KOH and 8 g/L phytic acid are shown in Figure 7. The Zn concentration at 40 s in anode area, the middle area and cathode area were separately 1.43 ± 0.02, 1.43 ± 0.02 and 1.43 ± 0.01 g/L. At 120 s, the values were separately 1.40 ± 0.02, 1.38 ± 0.01 and 1.38 ± 0.01 g/L (Figure 7a). The higher Zn concentration at 120 s in anode area than that in cathode area suggested that electromigration may induce Zn ions to be incorporated into MAO coatings. However, the P concentrations at 40 s in anode area, the middle area and cathode were separately 1.14 ± 0.01, 1.16 ± 0.01 and 1.17 ± 0.01 g/L. At 120 s, the values were separately 1.18 ± 0.01, 1.18 ± 0.01 and 1.20 ± 0.01 g/L (Figure 7b). Regardless of 40 s or 120 s, the P content in anode area was lower than those in cathode area (Figure 7b).

## 4. Discussion

In order to elucidate the influences of the four factors on the Zn and P amounts, the formation process of anodic coatings is deduced according to the property of the used electrolytes, the characteristics of MAO treatment and the coating performance.

### 4.1. How Zn Enters MAO Coatings

Anions and cations in water solution should arrive first at the anode/electrolyte interface and then enter into MAO coatings [16]. Anions can move to the anode by diffusion and electromigration, while cations usually move to the anode by diffusion [19,20]. With the prolonging of treating time, the developed electric field becomes stronger, and electromigration can play an important role in driving the anions toward the anode. During MAO, the ion concentration in the anode area is determined by its reaction speed into MAO coatings and the moving speed toward the anode. If the reaction speed is faster than the moving speed, the ion concentration in the anode area is lower than in the cathode area. In contrary, the ion concentration in the anode area is higher than in the cathode area when the reaction speed is slower than the moving speed.

**Figure 8:**
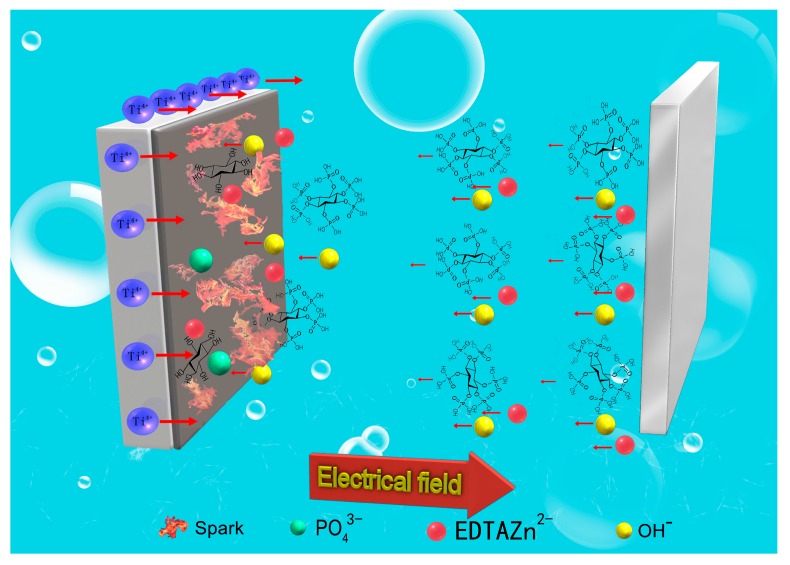
The schematic diagram showing the coating formation during MAO treatment.

According to the orthogonal experiment, EDTA-ZnNa_2_ concentration was not the first, but the third important factor on the Zn content of anodic coatings (Table 2). In addition, with the increasing EDTA-ZnNa_2_ concentration, the Zn content in MAO coatings did not continually increase. These results indicate that diffusion is not the main way of Zn ions into MAO coatings. According to ICP results shown in Figure 7a, at 40 s, the Zn concentration in anode area and cathode area did not exhibit significant differences. At 120 s, the Zn concentration in the anode area was larger than in the cathode area. This may have resulted from the properties of EDTA-ZnNa_2_ and phytic acid. In a water solution, EDTA-ZnNa_2_ can ionize into Na^+^ and negatively charged EDTA-Zn^2−^. Therefore, EDTA-Zn^2−^ can move toward the anode by diffusion and electromigration [17], as shown in Figure 8. In addition, as an organic macromolecule with 24 oxygen atoms, 12 hydroxyl groups and six phosphate carboxyl groups, phytic acid has powerful chelating capability with divalent and trivalent minerals [21,22]. Zn ions can also combine with phytic acid radicals to become negatively charged. Under the stronger field resulting from the prolonging of treating time (Figure 7a), electromigration but not diffusion, becomes the main method for Zn ions to integrate into MAO coatings. The results of the higher Zn concentration in the anode area than in the cathode area (Figure 7a) and the Zn content in MAO coatings determined by phytic acid concentration (Table 2) indicate that the reaction speed of Zn combined with P, not its moving speed, mainly influences the Zn amount in MAO coatings.

### 4.2. How P Enters the MAO Coatings

During MAO, the P content in anode area was lower than that in cathode area (Figure 7b). The reason may be pertinent to the property and structure of phytic acid. On the one hand, phytic acid is favorable for coating formation (Figure 4), and its reaction speed into anodic coatings may be fast during MAO. On the other hand, as a ringed carbohydrate with six phosphate groups, the moving speed of phytic acid toward the anode may be slow. Therefore, compared to Zn ions, electromigration plays a weaker role in driving P ions toward the anode. Under the fast reaction speed of phytic acid into MAO coatings and the slow moving speed toward the anode, the P concentrations in the anode area were lower than those in cathode area (Figure 8).

As an organic substance, phytic acid radicals are not stable and can be hydrolyzed into small molecular inositol phosphates in the temperature range between 320 and 345 °C [23]. It is believed that the instantaneous temperature on the anode surface during MAO can reach 2000 °C due to spark discharge [24]. Under the instantaneous high temperature on the anode, phytic acid radicals adjacent to the anode are hydrolyzed into inorganic phosphates, as shown in Figure 8. During MAO, the following reactions on the anode may take place [10]:

Ti − 4e^−^ = Ti^4+^ (1)

H_2_O = 2H^+^ + O^2−^ (2)

Ti^4+^ + 2O^2−^ = TiO_2_ (3)

H_3_PO_4_= 3H^+^ + PO_4_^3^^−^ (4)

3Zn^2+^ + 2PO_4_^3−^ = Zn_3_(PO_4_)_2_ (5)

**Figure 9:**
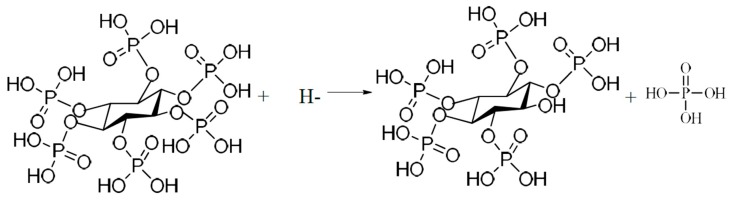
The first hydrolysis step of phytic acid during MAO.

**Figure 10:**
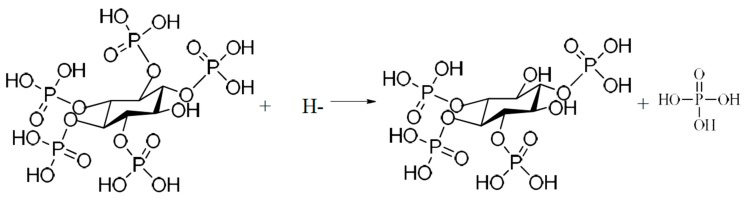
The second hydrolysis step of phytic acid during MAO.

Anatase and rutile TiO_2_ are acquired in anodic coatings during MAO treatment by Equations (1)–(3). According to Figure 9 and Figure 10 and Equations (4) and (5), Zn and P are incorporated into MAO coatings and their contents increase with the increasing phytic acid concentration. Meanwhile, Zn and P present the similar influencing regularity due to the formation of Zn_3_(PO_4_)_2_ in MAO coatings (Figure 3).

## 5. Conclusions

The influencing regularity of processing factors on Zn and P contents were investigated by using the orthogonal experiment of four factors with three levels in a solution containing EDTA-ZnNa_2_, KOH and phytic acid. Some conclusions are drawn as follows.

(1) Zn element in MAO coatings combined with P element exists in the form of Zn_3_(PO_4_)_2_. Zn enters into MAO coatings with P from phytic acid, while P takes part in coating formation mainly by diffusion.
(2) The impact of factors on the Zn content in MAO coatings is as follows: phytic acid concentration > KOH concentration > EDTA-ZnNa_2_ concentration > treating time, while the influencing order on the P amount is phytic acid concentration > KOH concentration > treating time> EDTA-ZnNa_2_ concentration.
(3) Phytic acid is beneficial to the development of MAO coatings, while NaOH inhibits the coating formation. With the increase of phytic acid concentration or the decrease of KOH concentration, the contents of Zn and P in MAO coatings present similar increasing tendencies.

## Figures and Tables

**Table 1:** Factors and levels of the orthogonal experiment.

Levels	Factors			
	EDTA-ZnNa_2_ Concentration (g/L)	KOH Concentration (g/L)	Phytic Acid Concentration (g/L)	Treating Time (min)
1	2	2	2	2.5
2	6	5	5	3
3	10	8	8	3.5

**Table 2:** The orthogonal experimental array and experimental results.

Experiment No.	EDTA-ZnNa_2_ Concentration (g/L)	KOH Concentration (g/L)	Phytic Acid Concentration (g/L)	Treating Time (min)	Zn (wt %)	P (wt %)
No.1	2	2	2	2.5	0.89	3.70
No.2	2	5	5	3	3.16	7.27
No.3	2	8	8	3.5	2.86	6.88
No.4	6	2	5	3.5	5.83	12.14
No.5	6	5	8	2.5	7.73	9.89
No.6	6	8	2	3	0.18	0.15
No.7	10	2	8	3	8.81	14.10
No.8	10	5	2	3.5	2.36	1.93
No.9	10	8	5	2.5	0.48	0.96
K_1_	6.91 (17.85)	15.53 (29.94)	3.43 (5.78)	9.1 (14.55)		
K_2_	13.74 (22.18)	13.25 (19.09)	9.47 (20.37)	12.15 (21.52)		
K_3_	11.65 (16.99)	3.52 (7.99)	19.4 (30.87)	11.05 (20.95)		
Difference	6.83 (5.19)	12.01 (21.95)	15.97 (25.09)	3.05 (6.97)		
Rank	3 (4)	2 (2)	1 (1)	4 (3)		
